# Capturing genomic relationships that matter

**DOI:** 10.1007/s10577-016-9546-4

**Published:** 2017-01-11

**Authors:** Cameron S. Osborne, Borbála Mifsud

**Affiliations:** 1 Department of Medical and Molecular Genetics, King’s College London, London, UK; 2 William Harvey Research Institute, Queen Mary University London, London, UK

**Keywords:** genome organization, promoter-enhancer interactions, Capture Hi-C, Hi-C pipelines

## Abstract

There is a strong interrelationship within the cell nucleus between form and function of the genome. This connection is exhibited across multiple hierarchies, ranging from grand-scale positioning of chromosomes and their intersection with specific nuclear functional activities, the segregation of chromosome structure into distinct domains and long-range regulatory contacts that drive spatial and temporal expression patterns of genes. Fifteen years ago, the development of the chromosome conformation capture method placed the nature of specific, long-range regulatory interactions under scrutiny. However, its development and integration with next-generation sequencing technologies has greatly expanded the breadth and scope of what is detected. The sheer scale of data offered by these important advances has come with new and challenging bottlenecks that are both experimental and bioinformatical. Here, we discuss the recent and prospective development and implementation of new methodologies and analytical tools that are allowing an in-depth, yet focussed characterisation of genomic contacts that are associated with functional activities in the nucleus.

## Main text

Within our genomes, genetic elements such as gene promoters engage in a multitude of relationships. Specific interactions to direct gene activity are formed with distal regulatory elements, such as enhancers. These elements can be positioned at very large distances from their interacting targets, yet communicate with each other by looping out intervening sequence and engaging in direct contact, mediated by the transcription factors they bind (Garcia-Gonzalez et al. 2016). Furthermore, chromosomes are demarcated structurally into topologically associated domains (TADs) that act to curtail the contact to within each of these regions (Dixon et al. 2012; Nora et al. 2012; Gonzalez-Sandoval and Gasser 2016). Finally, genes and other genetic elements may converge on one of the many sub-compartments that exist within the nucleus to carry out a particular function. For instance, multiple genes are transcribed concurrently at shared sites, called transcription factories (Osborne et al. 2004; Cook 2010). Whilst co-transcription at shared factories is not strictly predetermined, a preference exists that means some genes end up together more than expected, driven in part by co-dependence on certain transcription factors (Osborne et al. 2007; Schoenfelder et al. 2010; Hakim et al. 2013). No clear evidence suggests direct interaction between co-transcribing genes, yet they are likely to hold influence on each other.

For a proper understanding of how a gene is regulated, it is crucial to acquire a comprehensive list of all regulatory interactions that direct its activity. This is by no means a trivial matter. Genes often are influenced by multiple regulatory elements that may be scattered across a wide area surrounding the gene and interspersed by other genes and their elements. Added to this challenge, many appear to only exert their influences under certain circumstances, such as cell-type and temporal specificities, or in response to extra-cellular signalling. Characterisation of epigenetic signatures may help to identify regulatory elements and indicate the circumstances in which they are active, although evidence suggests that we still lack a complete syllabus of element types (Pradeepa et al. 2016). But more crucially, these signatures provide few clues as to the targets upon which they act.

Without any robust predictive measures, we are reliant on direct evidence of genomic interactions. For several decades, fluorescence in situ hybridisation microscopy has been used extensively to measure distances between loci, as well as positions with respect to nuclear space and functional compartments. Expansion of available fluorescent probes for labelling multiple nuclear components and loci has made it possible to carry out multidimensional analyses in single cells. However, a prescient inclination of relationships between elements is needed for probe targeting, and despite the emergence of ultra-high-resolution microscopes and smaller, brighter probes, specific genome interactions are difficult to discern. Moreover, some interactions may occur only rarely or transiently and thus present in a very small sub-population of cells, at any given time.

The past 15 years have seen the emergence of a new technique, developed to probe chromosome structure and genomic contacts. Chromosome conformation capture (3C) and a series of derived methods use a biochemical approach to provide a relative measure of proximity between genomic loci (Dekker et al. 2002; Lieberman-Aiden et al. 2009; Splinter et al. 2012). The underlying basis involves chromatin fragmentation of formaldehyde-fixed nuclei, usually by restriction enzyme digestion, followed by ligation that permits cross-linked fragments to be joined together. Close proximity favours cross-linking, which in turn favours their ligation. Whilst every fragment in each nucleus can ligate to a maximum of two other fragments, typically, libraries are generated from millions of cells and thus are comprise of a rich mixture of ligation events that reflect the proximity repertoire of every fragment across the whole genome.

The original 3C method was restricted to PCR detection of specific pairings of fragments, which limited detection of contacts with some prescient knowledge. However, the rapid and universal emergence of next-generation sequencing (NGS) technologies has opened up the method to successive adaptations to cast an increasingly wide net over the full scope of contacts. Arguably, the most versatile method to emerge to date is Hi-C (Lieberman-Aiden et al. 2009). By insertion of a biotin DNA tag at each ligation junction, the ligated genomic mixture can be sonicated into small, sequence-sized fragments and enriched by streptavidin affinity to select only the small fragments that contain a bone fide ligation junction. This step greatly reduces library complexity and ensures little wastage on sequencing fragments that lack a ligation junction. Sonication ensures distinctive ends to each ligation junction fragment, so sequenced duplicates that result from library PCR amplification can be discerned from unique ligation events and disregarded. Equipped with these features, and assayed in millions of cells, Hi-C libraries provide unparalleled scope and depth of measured ligation events across the entire genome.

NGS analysis of Hi-C libraries has shown that the majority of ligation events originate from the tight physical linkage of fragments that reflect their close positioning in the linear DNA sequence; contacts between fragments from the same stretch of DNA are highly represented, and typically, there is an inverse relationship between ligation frequency and linear separation. Yet, linearly separated fragment pairs may also have higher than expected ligation frequencies. These are usually the most informative events and can provide information on chromosome structure as well as interactions between elements such as gene promoters and enhancers.

Probably, the most conspicuous observation to materialise from Hi-C studies has been the existence of a structural compartmentalisation across chromosomes into TADs (Dixon et al. 2012; Nora et al. 2012). Each of these regions is flanked by boundary sequences that constrain intermingling of its chromatin to its own neighbourhood. Some TAD boundaries appear to be robust and fixed across cell types, and even species, and therefore likely represent larger structural constraints, which do not reflect regulatory functions. Others exist at the level of sub-TADs and may be responsive to different cellular conditions. These TAD structures appear to have an important role in maintaining regulatory environments; perturbation of the boundaries can lead to gene dysregulation in cancer and other diseases (Lupianez et al. 2015; Hnisz et al. 2016; Symmons et al. 2016; Taberlay et al. 2016).

Hi-C has been proven well suited for defining structural features such as TAD domains, which are stretched across hundreds of kilobases, yet it has been proven more challenging to delve deeper and attain sufficient resolution to detect specific contacts between discrete genomic regions, such as regulatory elements. This level of detail is buried within Hi-C libraries but often obscured by the immense complexity, with reads naturally distributed widely across the genome. Very deep sequencing is required to register enough reads for any given fragment to identify specific genomic contacts with confidence. Whilst this is not impossible, it is not cheap; Rao and colleagues resolved specific contacts to a resolution of 1 kb by sequencing a Hi-C library to a depth of 3.2 billion reads, which well exceeds the capacity than an entire Illumina HiSeq flow cell (Rao et al. 2014). Since most interest in high-resolution contact information is centred on gene regulation, it is likely that the majority is not immediately informative, as it will be collected from genomic regions with undetermined regulatory potential.

Methods have been developed to direct sequencing power toward sub-sets of the genome for NGS. Solution hybridisation selection employs a biotinylated RNA bait library, in vitro transcribed from a specially designed oligonucleotide array, to differentially enrich a NGS library (Gnirke et al. 2009). Upon hybridisation to the RNA baits, the targeted sequencing templates are immobilised on streptavidin-coated magnetic beads and isolated from off-target sequences. Most notably, this tactic has been applied to enrich whole genomic sequencing libraries for the 1–2% of the genome that comprises exonic sequences and greatly increase the coverage over coding regions.

Recently, several studies have applied library enrichment strategies to Hi-C to maximise the sequencing depth of contact information for a sub-set of genomic regions(Dryden et al. 2014; Jager et al. 2015; Ma et al. 2015; Martin et al. 2015; Mifsud et al. 2015b; Sahlen et al. 2015; Schoenfelder et al. 2015b; Ramani et al. 2016; Wilson et al. 2016). Whilst the Capture Hi-C (CHi-C), Hi-Cap and targeted DNase Hi-C studies have used different approaches to fragment the genome prior to ligation, they are similar in their use of RNA enrichment baits directed to the ends of targeted fragments to enrich for the ligation events that they form. A similar strategy has been used in Capture-C, which enriches specified contacts from 3C libraries, rather than Hi-C, using DNA enrichment baits (Davies et al. 2016). In our own CHi-C studies, we targeted approximately 22,000 gene promoter-containing fragments, representing less than 6% of the genome, for selection (Mifsud et al. 2015b; Schoenfelder et al. 2015a). By this strategy, we obtained a tenfold enrichment of read depth for targeted fragments. In other terms, sequencing of a CHi-C library on a single lane of a flow cell delivers more reads for these genome-wide regions of interest than a Hi-C library sequenced on an entire flow cell. With augmented read depth, one can think of these experiments as quantitative, massively parallel 4C studies and can begin to properly interrogate the genomic contacts made by these elements in a cost-effective manner.

Successful capture of Hi-C libraries requires careful consideration to ensure high enrichment of the targeted fragments. However, it is typical to experience orders of magnitude differences in fragment enrichments in Hi-C libraries. Many of these disparities are innate and unavoidable, relating to the hybridisation characteristics of the targeted sequences, although careful experimental design may help to mitigate these effects. As with all capture applications, the balance of GC content and uniqueness of sequence directs the hybridisation efficiency of any given RNA bait. These frustrating traits can be hard to avoid for certain target fragments; in our experience, relaxation of GC content criteria (range of 35–60%) to force RNA baits into fragments generally leads to disappointing results. In these cases, a best solution might be to tile multiple sub-optimal RNA baits within these regions. Obviously, targeting both ends of a fragment for capture, if possible, can mitigate the impact of poorly capturing RNA baits and improve the likelihood for efficient enrichment. It is also best practise to direct the RNA bait as close as possible to the end of the target fragment, adjacent to the ligation junction, since the bait target sequence is more likely to be decoupled from the ligation junction during sonication if it is positioned further away. This creates inherent challenges for the targeted DNase Hi-C capture method, which uses random DNaseI treatment to fragment the genome (Ma et al. 2015). With no set point of digestion, RNA capture baits must be tiled across a region of interest to ensure sufficient enrichment for a DNA element.

Composition of the library to be captured can also impact the success of enrichment. Hi-C libraries are naturally more efficient to capture, compared to 3C libraries. Bone fide ligation junctions are marked by the insertion of a biotin mark in Hi-C libraries, which is used to enrich the libraries prior to RNA bait capture. In effect, a Hi-C library is composed solely of ligation junctions. By contrast, in 3C libraries, no such enrichment occurs. The significance of this is twofold; firstly, the 3C library retains the complexity of a full genome, which likely provides greater competition for hybridisation of the RNA baits. Secondly, restriction enzyme digestion in formaldehyde-fixed nuclei is not particularly efficient, ranging between 70 and 80% for a typical restriction enzyme site; meaning that 20 to 30% of every fragment junction have not undergone ligation. Without selection of real ligation events via a biotin mark, these non-informative, non-digested events are also sequenced, consuming value capacity. Notably, the 3C-based method Capture-C yields a return of less than 3% on-target, unique, sequence reads, compared to 34–48% by CHi-C (Mifsud et al. 2015b; Davies et al. 2016). A captured 3C library is indeed easier and faster to prepare, but it appears to come at considerable cost during the sequencing.

The choice of method by which the genome is fragmented will influence the resolution at which contacts are detected. Both six-cutter and four-cutter restriction enzymes have been used successfully (Mifsud et al. 2015b; Sahlen et al. 2015). The commonly used six-cutter enzyme, HindIII, segments the human genome into a median fragment size of 2.3 kb. Clearly, the more frequent rate of cleavage offered by four-cutter enzymes can offer better resolving power than six cutters. This is advantageous for discerning contacts that occur over shorter distances. However, higher resolution is likely offset by efficiencies of ligation and target enrichment. Genomic fragments that are shorter than 1 kb ligate with poorer efficiency than fragments between 1 and 10 kb (Naumova et al. 2012)*.* Furthermore, the increased cutting rate places greater restriction on where the capturing RNA bait can be placed. These limitations may not hinder detection of strong contacts, but those that are less robust appear to escape detection. Neither four nor six cutters can circumvent the fact that fragment size is variable, thereby limiting the resolution, depending upon the location. In this regard, the random nature of DNaseI treatment seems advantageous, albeit with the caveats regarding RNA bait placement, as described above.

Good candidates of fragments engaged in direct interactions should stand out by the strength of ligation frequency, but this is not always straightforward to interpret. Hi-C and related methods are not direct measures of interactions nor strictly speaking, a direct measure of proximity. Rather, they provide a relative measure of proclivity for ligation between pairs of DNA fragments. Clearly, fragments that are close to each other are more likely to ligate together than others that are far apart. However, competition is an important influence on ligation frequency. Since each fragment end can ligate to just one other fragment, to which fragment it will ligate depends heavily upon the relative positions of potential suitors; tight proximity of one interacting fragment will dampen ligation to other interacting fragments that are not nearly as close. The position of the interaction in relation to the fragment end will also be influential. The number of ligation-ready fragment ends in proximity can vary, since a DNA stretch of 10 kb may have a single-restriction site or it may have several. Diverse states of chromatin compaction could also conceivably alter the number of fragment ends within the immediate vicinity. Also, as Hi-C experiments are carried out on large cell numbers, a close interaction that occurs in half the cells can appear equivalent to a looser association that occurs in all cells. An impact of these variables is that single change in the composition of a fragment’s immediate environment can alter the perception of all its interactions, which is an important consideration when using these methods to compare different cell types or conditions.

Beyond the information on direct interaction, other spatial information is embedded within Hi-C libraries. In addition to the TAD structure of chromosomes discussed above, we have detected co-association of groups of genes positioned across several chromosomes, including Polycomb group-associated genes, and genes encoding histones and zinc-finger proteins (Mifsud et al. 2015b). These clusters are not typically detected at single-restriction fragment resolution and likely do not imply specific interaction, but rather a co-association at a nuclear sub-compartment, such as a transcription factory. The functional consequence of such co-association is unclear, although it may influence rates of transcription by providing a permissive environment (Kang et al. 2011). Significant improvements to the Hi-C methodology to reduce spurious ligation events that create background noise will likely aid the exposure of more of these relationships.

Even with all these methodological limitations, careful, deliberate processing and analysis can yield highly useful information on genome organisation. The technical constraints described above should be considered when interpreting the data, but they cannot be analytically resolved. However, a number of experimental biases and artefacts can be reconciled and corrected, and several specialised pipelines have been developed to make sense of the data.

The first step is to reduce the Hi-C libraries to include only meaningful reads. Hi-C libraries contain a highly variable number of non-informative reads, which if included would compromise analyses. These need to be removed before any downstream analyses can take place. HiCUP is a widely used pipeline that both maps reads to the reference genome and removes non-informative read pairs and artefacts (Wingett et al. 2015). It removes read pairs that map to adjacent fragments or span multiple adjacent fragments but are smaller than the size selection limit at library preparation, as these could come from re-ligation. HiCUP also filters out any read pairs that map to the same restriction fragment, as these represent self-ligations or non-ligated DNA fragments, and read pairs where the theoretical insert size is smaller or larger than what is expected from size selection, as these are likely to represent incorrect mapping. The final filtering step is to remove exact duplicates, as at current sequencing depths, we do not expect to see duplicated read pairs due to biological reasons but rather due to PCR amplification artefacts.

Whilst this Hi-C data pre-processing and use of HiCUP have become standardised, there is considerably more controversy surrounding the downstream methods used for data normalisation and interaction calling. Several alternate pipelines have been developed, and whilst there is a reasonable rationale for each, there is no perfect pipeline.

Hi-C libraries contain multiple biases that can skew the interpretation of the data. Analysis pipelines are designed to normalise for their effects. These biases include PCR amplification biases, which arise due to differences in the amplified DNA sequence, determining the kinetics of denaturing and annealing at every PCR cycle. Generally, GC-rich regions, though not extremely GC-rich regions, are preferentially amplified. However, the exact bias depends on the temperature profile, the polymerase and the buffer used in the PCR reaction. Since the amplified DNA fragments in a Hi-C or Capture Hi-C experiment are the ligated ends of restriction enzyme fragments, the effect of the PCR bias is dependent on the GC content only proximal to the restriction fragment ends. The distance from the restriction site where the GC content matters is dependent on the fragment size distribution after sonication.

An additional bias that can result in either under- or overrepresentation of a given restriction fragment in the Hi-C or Capture Hi-C dataset relates to the mappability of the restriction fragment, again within the 100–800-bp region surrounding the restriction site. Only uniquely mapping read pairs are considered in the analysis of such data, and therefore those restriction fragments, where the end is repetitive will be underrepresented in the read pool.

Whilst these biases are in common with other NGS libraries, a bias that is specific to chromosome conformation capture libraries such as Hi-C or Capture Hi-C is the restriction fragment length. Very short or very long fragments are ligated at lower efficiency, whereas fragments of similar sizes are most effectively ligated together (Yaffe and Tanay 2011). In addition, fragment size also has an effect when the analysis is carried out at lower resolution. The larger the fragments are in a fixed size region, the fewer possibilities the region has to form a ligation product, which results in underrepresentation of the region.

An additional layer of bias is associated with Capture Hi-C and relates to the enrichment step. RNA baits will hybridise to their target sequences with varying efficiencies, which affects the enrichment efficiency and leads to unequal numbers of reads for each targeted fragment and the ligation events with which they are involved.

To eliminate these biases, analytical pipelines have been developed that employ two main normalisation tactics. One approach models the effects of each bias separately and has been taken by the hicpipe (Yaffe and Tanay 2011) and HiCNorm (Hu et al. 2012) pipelines, applied to Hi-C libraries. Whilst it has not been used in the analysis of Capture Hi-C libraries, these models could be extended with the inclusion of an extra variable to normalise for capture efficiency. This method does work well; however, other potentially unappreciated biases will not be accounted for.

The second approach takes an agnostic view of biases and assumes that each that is present in the experiment will affect the general “visibility” of the given fragment or region. Therefore, biases can be eliminated by use of a visibility score, the total number of reads that map to a given region across all of its interactions, as a correction factor. This concept is used by many of the current Hi-C analysis methods, such as hiclib (Imakaev et al. 2012) and HiC-Pro (Servant et al. 2015), which apply it to their matrix-balancing normalisation algorithm. These methods are not applicable to Capture Hi-C data, because they assume that the visibility of every fragment should be the same, which is not the case when the matrix contains both baited and non-baited fragments. The GOTHiC pipeline uses a cumulative binomial distribution, which assumes that the visibility scores of two interacting fragments affect the observed read count between those two regions independently, in a multiplicative manner (Mifsud et al. 2015a). This principle holds true for Hi-C and for contacts between a baited and a non-baited fragment in Capture Hi-C. However, it is problematic for ligation pairs between bait-targeted fragments that happen to ligate together. In these “bait-bait” events, either or both sides of the ligation pair can be pulled down, and hence, there is an interdependence of the two fragments’ visibility, having both a multiplicative and an additive component. To reconcile this, an alternative version of GOTHiC that accounts for bait-to-bait ligation products has been developed (Mifsud et al. 2015b; Schoenfelder et al. 2015b). In general, these visibility-based methods also perform well, like the explicit bias correction methods (hicpipe and HiCNorm). However, it assumes that every region should have the same visibility and does not account for the possibility that some regions may be highly represented due to being an interaction hub.

To establish which regions are in close proximity in a cell population, one must move beyond the removal of the effects of different biases and apply a statistical test to determine whether the observed ligation frequencies are due to high physical proximity in vivo or due to rare collisions and random ligations.

GOTHiC is one of the few pipelines that separate interactions into those statistically significant, reflecting a physical proximity in vivo, and those that are random. It uses the visibility of the interacting fragments to calculate the number of reads expected between the two regions, and then with a cumulative binomial test, determines whether the observed numbers of reads are significantly higher than expected. By this way, it both removes biases and separates interactions, discerning real proximity versus random collisions in both Hi-C and capture Hi-C datasets.

In vivo proximity can either reflect a 1D relationship, from being relatively close to each other on the DNA molecule, or a specific 3D interaction loop. Most contacts observed occur due to the interacting fragments’ relative position on the DNA polymer; being on the same molecule imposes upon them a physical constraint. In all Hi-C-type experiments, a declining number of reads is observed, as the genomic distance between the two interacting regions is increased.

In some analyses, a rationale is applied that contacts need to be normalised for distances that separate the fragments, based upon the logic that a true regulatory interaction will naturally occur more often than other contacts of the same distance. There are a number of methods that apply this idea for Hi-C, e.g. Fit-Hi-C (Ay et al. 2014), HOMER (Heinz et al. 2010) and HiFive (Sauria et al. 2015). For Capture Hi-C, so far, there is only CHiCAGO (Cairns et al. 2016) that uses distance correction, taking into account the different properties of bait-bait and bait-non-bait interactions.

Whilst the rationale for a distance correction is valid, it has certain limitations and a one-size-fits-all approach is likely to miss or miscall interactions. Firstly, it is almost impossible to discern specific interactions if the original distance is very short, since there will be high contact even between non-specifically interacting regions; the increased contact of specific interactions will not be significantly higher. Secondly, distance correction makes assumptions based upon the average signal decay for all fragments across the entire genomic dataset. However, there is considerable variability in terms of directionality, with the reads of some fragments being skewed toward one direction or another, which can result from relative positioning to TAD boundaries. The degree of spread of reads from a fragment may also vary, with the reads of some fragments being concentrated very nearby, with other extending to considerable distances; this effect may in part be due to differences in chromatin compaction. The final complication is that distance correction requires the correct genome build for mapping. This is not so much an issue in healthy cells; however, diseased states are often associated with genomic rearrangements, such as cancers. As cancer cells and cell lines often have multiple and complicated heterogeneous rearrangements, genome build correction is not a straightforward problem.

Whilst distance correction remains difficult to implement properly, it may be advisable to avoid it and instead infer real interaction through overlay of functional profiles using other datasets, such as ChIP-seq. Yet, even this is limiting; many active promoters interact with distal regions that do not contain the widely accepted enhancer-like histone modification signature (Mifsud et al. 2015b). At least, some of these interactions are functional. Recent evidence highlights the existence of other classes of enhancers that do not have the canonical signature (Pradeepa et al. 2016). Moreover, there is no consensus signature for other types of elements, such as silencers. Therefore, there is still some way to go in the use of functional signatures to provide context of interactions.

An alternative analysis aims to identify functional interactions by characterising differential contacts between two cell types or conditions. HOMER, HiBrowse and diffHiC were developed for detecting differential interactions in HiC data, built upon previous methods that were originally applied to identify differentially expressed genes in RNA-seq data (e.g. edgeR). The framework has been used for captured data, using another differential expression method (DeSeq2) for NG Capture-C (Davies et al. 2016). However, as discussed above, small changes in the direct environment of a region can have an effect on the detection of its other, constant interactions. Differential ligation efficiency between two fragments therefore does not necessarily reflect differential interaction.

Finally, put in the context of epigenetic marks, RNA expression and other functional genomics data, Hi-C and capture Hi-C contacts are rich for exploration and interpretation, with the possibility of zooming in to different windows in the genome. Several bioinformatic packages have been developed for this purpose, such as Sushi (Phanstiel et al. 2014), JuiceBox (Durand et al. 2016a) and HiCDat (Schmid et al. 2015). With the Sushi R package, genomic interaction data, including Capture Hi-C data, can be plotted along ChIP-seq, RNA-seq and annotation tracks. JuiceBox enhances exploration of a number of previously published Hi-C data and other Hi-C sets processed by Juicer (Durand et al. 2016b). It enables visualisation of contact matrices with several normalisation methods at different resolutions; highlights 2D structures in the data, such as interaction domains and loops; and aligns other types of data, e.g. histone modification and transcription factor-binding profiles. HiCDat can compare samples and finds significant correlation or enrichment with other types of data. There is a quickly increasing number of pipelines and visualisation tools for genomic interaction data; however, to date, there are only a few applicable to Capture Hi-C. One that was specifically developed for Capture Hi-C is the Capture Hi-C plotter, which shows contacts on a per-bait circos diagram and annotates the interacting fragments (Schofield et al. 2016).

## Perspective

With the ever-increasing sequencing capacity of NGS technologies, it may become cost-effective to bypass a capture step for Hi-C libraries and still attain a high-resolution map of genome-wide contacts. However, access to the computational power for processing plus the limitations of storage of such enormous and complex datasets will not diminish and will likely become the major bottleneck.

To date, most studies that have employed a capture step to enrich a Hi-C library have focussed on the interactions that are made by promoters. This will continue to supply rich veins of data, as different cell types are assessed, different species are compared and response to different cell extrinsic influences are monitored. Diseases are generally reflected through global changes to the transcription programme. These are likely to be underpinned by alterations to the regulatory contacts that dysregulated genes make, whose characterisation may offer diagnostic and prognostic values, as well as new means of intervention to explore.

Beyond the interactome of promoters, capture Hi-C allows a great versatility to enrich for the interactions of other meaningful sequences. Already, GWAS SNPs have been enriched for capture to identify their functional targets (Dryden et al. 2014; Jager et al. 2015; Martin et al. 2015), and this direction will continue to reap rewards for disease association studies. Enrichment will also be directed to other genomic features, such as regulatory elements and structural components like TAD boundaries and lamina-associated domains.

Other forms of genomic organisation that are not mediated through direct interactions are also likely to come to the forefront. Regions across the genome coalesce at functional and structural sub-compartments for common purposes. With a looser association than a direct interaction through protein-protein contacts, their detection will likely be more challenging. Yet with more focussed enrichment, the function of many nuclear sub-compartments may be revealed.

Hi-C experiments have been limited to pair-wise analysis of contacts, generally in large cell populations. Whilst this is very useful at identifying which cohort of fragments interacts with a particular element, it is difficult to infer which sets of interactions occur in concert. Perhaps, this can begin to be addressed with longer sequencing reads to measure events where the two ends of a target fragment ligate to distinct partners, as a 4C study suggests (Jiang et al. 2016).

Despite the numerous pipelines and tools to analyse Hi-C type data, there is still scope for development. Hi-C analysis would benefit from locus-specific distance correction, new visualisation methods and statistically grounded integration of diverse genomic data. Using 3D structure models as backbones for diverse cell-type-specific genomic data could enhance data exploration. There is still a lack of tools that can accommodate the high coverage differences between baited and non-baited regions in Capture Hi-C data, as well as methods that can assess enrichments in these datasets.

Ultimately, the contact information provided by Hi-C methods, overlaid signatures of activity such as histone modifications, can infer a functional relationship. However, as always, the burden of proof requires these interactions to be tested directly. Recent adaptations to CRISPR technology provide high-throughput screens to assay function of interacting elements (Fulco et al. 2016; Sanjana et al. 2016). Full integration of such a potent arsenal of tools to measure both form and function will continue to probe the numerous activities of the nucleus.

## Abbreviations

NGS: Next-generation sequencing
TAD: Topologically associated domains
3C: Chromosome conformation capture
CHi-C: Capture Hi-C
